# Regulatory requirements for marketing fixed dose combinations

**DOI:** 10.4103/2229-3485.71768

**Published:** 2010

**Authors:** B. G. Jayasheel

**Affiliations:** *Bangaragiri, #7368, 7^th^ Cross, Ashok Nagar, Tumkur - 572103, India*

**Keywords:** Clinical trials, fixed dose combinations, India, marketing approval, regulatory

## Abstract

The development of fixed-dose combinations (FDCs) is becoming increasingly important from a public health perspective. FDCs have advantages when there is an identifiable patient population for whom treatment with a particular combination of actives in a fixed ratio is safe and effective and when all of the actives contribute to the overall therapeutic effect. Such combinations of drugs are particularly useful in the management of chronic diseases. In addition, there can be real clinical benefits in the form of increased efficacy and/or a reduced incidence of adverse effects. Additional advantages of FDCs are potentially lower costs of manufacturing compared to the costs of producing separate products administered concurrently, simpler logistics of distribution and reduced development of resistance in the case of antimicrobials. Above all, FDC therapy reduces pill burden and improves medication compliance. Although, FDCs seem to be ideal under certain pre-defined circumstances, if a dosing adjustment is warranted, there may not be an FDC available in the most appropriate strength for the patient and if an adverse drug reaction occurs from using an FDC, it may be difficult to identify the active ingredient responsible for causing the reaction. Appendix VI of Schedule Y (Drugs & Cosmetics Rules 1945, India) states the requirements for marketing approval of various types of FDCs. The same is further elaborated in this article to provide a detailed guidance including the clinical trial requirements. However, the heterogeneity of the therapeutic field makes it difficult to develop a standard guidance document.

## INTRODUCTION

A fixed-dose combination (FDC) is a formulation of two or more active ingredients combined in a single dosage form available in certain fixed doses. A ‘combination pack’ consists of more than one medicinal product, marketed with a single name and package (e.g. box, blister pack) and are intended for simultaneous or sequential administration.[1] This article focuses only on FDCs.

The development of FDCs is becoming more common either to improve compliance or to benefit from the added effects of the two or more active drugs given together. They are being used in the treatment of a wide range of conditions and are particularly useful in the management of chronic conditions. Each dose combination should be carefully justified and clinically relevant (e.g. in cases when each component of the FDC has several possible dosages, dosages that have shown benefit on clinical outcomes may be preferable).

## INDIAN SCENARIO

Appendix VI of Schedule Y (Drugs & Cosmetics Rules 1945, India) provides details about the requirements for manufacture/import approval and marketing of various types of FDCs.[2] The discussion is restricted to approval of FDCs as a finished pharmaceutical product.

In general, a clear justification with a valid therapeutic rationale of the particular combination of active substances proposed will be the basis of approval by regulatory authorities. It is *not always* necessary to generate new (*original*) data. Evidence may be obtained from the *scientific literature*, subject to its being of adequate quality. An application for a marketing authorization of FDCs may comprise entirely original data, entirely data from the literature or both original data and data from the literature (“*hybrid*”). It is likely that *hybrid* submissions will be the most common type. Chemical and pharmaceutical data should be always totally original, unless there is sufficient justification with literature when partial data can be in-original.

When a clinical trial (CT) is required, confirmatory studies to prove efficacy, preferably by parallel group comparisons in which the FDC is compared to its individual substances, may be considered. When feasible, a placebo arm may be incorporated. Comparative CTs of the FDC with reference treatment may be necessary, especially when the therapeutic justification talks more on the FDCs superiority over a reference treatment.

## CATEGORIZATION AND DATA REQUIRED FOR APPROVAL OF FIXED-DOSE COMBINATIONS

### Broad classification

FDCs can be broadly divided into following groups:

- FDC not marketed in India and one or more active ingredient(s) is a new drug
- FDC not marketed in India but the active ingredients are approved/marketed individually and it is likely to have significant (pharmacokinetic/pharmacodynamic) PK/PD interactions. This can be further categorized into,
  - FDC marketed abroad
  - FDC not marketed anywhere but individual components used concomitantly
  - FDC not marketed and individual components are not used concomitantly
- FDC marketed in India but some changes are sought
- FDC - only for convenience
- Subsequent FDC approvals after the approval of primary applicant’s FDC in India

### General requirements

Generally following documents are required for manufacture/import and marketing approval of FDCs. However, additional data or documents may be required for some specific types of FDCs.

- Form 44
- Treasury challan of INR 15,000 if all active ingredients are approved in India for more than one year, or INR 50,000 in case any of the active ingredient is approved for less than one year. However, a challan of only 15000 is required, in case the applicant has already submitted an application along with a challan of 50000 toward any of the single active ingredient approval, which is less than one year old.
- Complete chemical and pharmaceutical data: Generic name(s), chemical name(s), physico-chemical properties, dosage form, composition, specifications and method of analysis of the finished product, validation of analytical methods, outline of the method of manufacture of finished products, stability data of the proposed dosage form, and dissolution studies data for all solid oral dosage forms.
- Rationale for combining them in the proposed ratio and therapeutic justification along with supporting literature.
- Summary of drug-drug-interactions (known and/or expected) among the active ingredients present in the FDC, *along with its implications*. This should be prepared and signed by a competent person on behalf of applicant.
- Clinical trials data showing safety and efficacy of the FDC in the same strength (that has been carried out in other countries, when applicable) including published data.
- In case of injectable formulation, sub-acute toxicity data conducted with the applicants’ product.
- The regulatory status of the FDC in other countries including countries where the drug is marketed, approved, approved as investigational new drug (IND) and/or withdrawn if any, with reasons, restrictions on use, if any in countries where marketed/approved, free sale certificate/certificate of pharmaceutical product from the country of origin (in case of import of the finished form of the FDC) and a copy package inserts, promotional literatures of FDC circulated in those countries where it is marketed.
- Source of bulk drugs/raw materials (for those active ingredients which are considered as new drugs) - If the applicant has a manufacturing license for that bulk drug(s), a copy of the same is required, or a consent letter from the approved source regarding supply of material may be produced, as the case may be. [Note: This is required when the applicant is manufacturing the FDC as finished formulation]
- In case if the applicant does not have an approval to manufacture any of the active pharmaceutical ingredient (API) which is considered as a new drug, applicant can either import the API (when the applicant has to submit all relevant documents and comply with further requirements for import of API) or manufacture the API (when the applicant has to submit all relevant documents and comply with further requirements for manufacture of API) or obtain the API from another manufacturer which is not yet approved by competent authorities (when the respective manufacturer of the API has to file an application separately in Form 44 along with treasury challan of requisite amount with all relevant documents). Parallel submission is recommended to save time as this can be processed simultaneously with the application for the FDC; however, the approval of the FDC will be considered after the approval of API.
- Copy of proposed package insert (generic name of all active ingredients; composition; dosage form/s, indications; dose and method of administration; use in special populations; contra-indications; warnings; precautions; drug interactions; undesirable effects; overdose; pharmacodynamic and pharmacokinetic properties; incompatibilities; shelf-life; packaging information; storage and handling instructions) and draft Label/ Carton etc.

### Specific requirements

The aforesaid first category of FDC includes those in which one or more active ingredient is a new chemical/ biological entity and the proposed combination is not available in India. For such FDCs to be approved for marketing, data to be submitted will be similar to data required for any new drug application including clinical trials as per Schedule Y of Drugs and Cosmetics Rules 1945.[2]

When the FDC is not marketed in India but the individual active ingredients are approved and the proposed FDC is likely to have significant PK/PD interactions, then no additional clinical trials are needed if the proposed FDC is marketed abroad or it is a common practice to use the individual components concomitantly. A summary of available pharmacological, toxicological and clinical data on the individual ingredients and any available clinical data showing safety and efficacy of the FDC/concomitant use of the individual active ingredients, in the same strength, including published data has to be provided. If enough supportive literatures are not available, then ‘*adequate evidence*’ on safe and effective concomitant use of the individual ingredients should be provided. This may be generated by means of taking opinion from subject experts, etc.

For any other such FDCs e.g. the FDC is not marketed anywhere in the world and the individual components are *not* concomitantly used in routine clinical practice and the ingredients are likely to have significant PK/PD interaction, clinical trials may be required. For obtaining permission to carry out clinical trials with such FDCs, a summary of available pharmacological, toxicological and clinical data including published data on the individual ingredients should be submitted, along with the rationale for combining them in the proposed ratio. In addition, acute toxicity data (LD 50) and pharmacological data should be submitted. Bioavailability/ bioequivalence (BA/BE) study protocol (when applicable), and clinical study protocol, patient information sheet and informed consent form, copy of ‘ethics committee’ approval letters (if available), case record form, undertaking by investigator(s) as per the appendix VII of Schedule Y of Drugs and Cosmetics Rules, 1945 and their curriculum vitae and a certificate of analysis of study drug(s) has to be submitted. After successful completion of clinical trial (BABE when needed), a study report has to be submitted to get the marketing approval for the FDC. The study report should be certified by each of the participating investigator(s) in the study and the certification should acknowledge the contents of the report, the accurate presentation of the study as-undertaken, and express agreement with the conclusions.

When the FDC is already marketed, but now it is proposed either to change the ratio of active ingredients or to make a new therapeutic claim, then, an appropriate rationale including published reports (if any) should be submitted to obtain marketing permission. Permission may be granted depending upon the nature of the claim and data submitted. A clinical trial/ BABE study may be required if the justification provided for the new claim(s) is not satisfactory.

When the FDC includes those whose individual active ingredients (or *drugs from the same class*) have been widely used in a particular indication(s) for years, their concomitant use is often necessary and no claim is proposed to be made other than convenience. It will have to be demonstrated that the proposed dosage form is stable and the ingredients are unlikely to have significant interaction of a PK/PD nature. No additional animal or human data are generally required for these FDCs, and marketing permission may be granted if the FDC has an acceptable rationale.

When an applicant submits an application for approval of FDC which is of the same strength/ ratio, formulation and indication(s) of the already approved FDC of a primary applicant (subsequent approvals), no BA/BE study is required unless any of the individual ingredients are approved for less than four years in India, as it is considered as a new drug. BE study is required to be conducted with the proposed FDC comparing with the individual ingredients administered subsequently.

### Points to remember

- All FDCs should be based on convincing therapeutic justification.
- An indication must be a well-recognized disease state, or two closely related diseases, or a modification of a physiological or dysfunctional state, or a syndrome or pathological entity.
- In a FDC, each active substance should make a contribution to the claimed effect or improve the overall benefit risk ratio by mitigating side effects.
- The product should be so formulated that the dose and proportion of each substance present is appropriate for the intended use.
- The possibility of interactions between the substances should always be considered. Appropriate data should be submitted either to establish that such interactions do not occur or that they are clearly recognized and defined.
- Global regulatory status of the FDC has to be made very clear in the application and highlighting the same in the covering letter of the application is recommended.
- When the addition or the potentiation of pharmacodynamic effects of the ingredients constitute the rationale of the FDC, several dose combinations for each substance might have to be tested and the concentration-response information can help to select the FDC leading to a satisfactory response.
- BA/BE study is required when any of the individual ingredients are approved for < 4 years in India, even when the FDC is available outside India.
- When the FDC corresponds closely to combinations that are already in widespread use, a well founded bibliographical data analysis could be submitted.
- In some cases, studies have to be specifically designed to determine the minimal effective dose and usual effective dose of the FDC. Multiple dose-effect studies may be required. The multilevel factorial design may be used.
- When an FDC is intended to relieve simultaneously different symptoms or to prevent different diseases, selected doses of each active ingredients are often those commonly used for the treatment of each symptom or the prevention of each disease.
- In the case that the doses used in the fixed combination are identical to the doses used in the broad clinical setting and safety data generated with these doses are available, demonstration of comparability in the PK properties might be sufficient.
- Confirmatory clinical trials are necessary to prove efficacy, preferably by parallel group comparisons in which the FDC is compared to its individual substances. Comparative clinical studies of the FDC versus reference treatment might be necessary in order to put into perspective the improvement obtained with the FDC.
- When the FDC is proposed to be used as second line therapy, a clinical trial in non-responders or patients insufficiently controlled with optimally-dosed-monotherapy is recommended.
- Specific disease-related scientific guidelines issued by any reputed organizations/ professional societies may be referred for designing the study.
